# Relationship between Zinc (Zn^**2+**^) and Glutamate Receptors in the Processes Underlying Neurodegeneration

**DOI:** 10.1155/2015/591563

**Published:** 2015-05-27

**Authors:** Bartłomiej Pochwat, Gabriel Nowak, Bernadeta Szewczyk

**Affiliations:** Department of Neurobiology, Institute of Pharmacology, Polish Academy of Sciences, Smetna 12, 31-343 Krakow, Poland

## Abstract

The results from numerous studies have shown that an imbalance between particular neurotransmitters may lead to brain circuit dysfunction and development of many pathological states. The significance of glutamate pathways for the functioning of the nervous system is equivocal. On the one hand, glutamate transmission is necessary for neuroplasticity, synaptogenesis, or cell survival, but on the other hand an excessive and long-lasting increased level of glutamate in the synapse may lead to cell death. Under clinical conditions, hyperactivity of the glutamate system is associated with ischemia, epilepsy, and neurodegenerative diseases such as Alzheimer's, Huntington's, and many others. The achievement of glutamate activity in the physiological range requires efficient control by endogenous regulatory factors. Due to the fact that the free pool of ion Zn^2+^ is a cotransmitter in some glutamate neurons; the role of this element in the pathophysiology of a neurodegenerative diseases has been intensively studied. There is a lot of evidence for Zn^2+^ dyshomeostasis and glutamate system abnormalities in ischemic and neurodegenerative disorders. However, the precise interaction between Zn^2+^ regulative function and the glutamate system is still not fully understood. This review describes the relationship between Zn^2+^ and glutamate dependent signaling pathways under selected pathological central nervous system (CNS) conditions.

## 1. Introduction

During recent years, our knowledge about the functioning of the glutamate system and its importance for the physiology of nervous system has significantly increased. Today, the role of glutamatergic pathways is not only considered in the context of the excitability of neurons. Our understanding of the physiological role of the glutamate system is much deeper and we can provide many data showing the involvement of the glutamatergic system in the regulation of very complex processes like neuroplasticity, cell death, cell survival, and many others [1–3]. Additionally, these discoveries may have practical significance, because we may associate dysfunction of these pathways with the development of many debilitating disorders, such as Alzheimer's disease, Huntington's disease, ischemic injury, epilepsy, schizophrenia, or depression [4]. Despite undeniable progress in our understanding of the pivotal role of glutamate system in the brain's functioning, there are still some issues that need clarification. One of the most fascinating issues is the significance of bivalent zinc ions (Zn^2+^) for the suitable action of the glutamate system and its role in the physiological and pathophysiological states of the brain.

The influence of Zn^2+^ on the structure of the cells and biochemical processes is very complex. Zn^2+^ is a ubiquitous trace element in the human body and the high concentration of Zn^2+^ is found in the brain [5]. Within brain, Zn^2+^ is nonuniformly distributed and it is most abundant in the hippocampus, amygdala, cortex, and olfactory bulbs. For example, in the hippocampus, a region of the brain essential for learning and memory, Zn^2+^ concentrations can reach up to 300 *μ*M in the mossy fiber boutons of neurons. Neuronal Zn^2+^ is partitioned into two main classes: a static and a labile Zn^2+^ pool [6]. Static Zn^2+^ pool plays an important structural function for the stability of great numbers of proteins and is a cofactor of about 300 enzymes which are engaging in the regulation of cellular processes and signal transduction pathways. It is worth mentioning that most of these processes are unspecific and apply to both nerve and other cells [7, 8]. However, Zn^2+^ also plays a role specific for the functioning of nerve cells. As has been shown in many studies, Zn^2+^ is stored in the synaptic vesicle in glutamate neurons (zincergic neurons) and released during neuronal activity into the synaptic cleft [9–11]. The exact amount of such release is controversial; some studies indicated that transient Zn^2+^ increases may reach 1–100 *μ*M, while other studies pointed to lower (submicromolar) concentrations [12]. These uncertainties arise from the fact that measurement of actual Zn^2+^ levels within the synaptic cleft is difficult, given the short time in which the free ion is present in the synapse [13].

In physiological conditions, Zn^2+^ modulates mainly postsynaptic, ionotropic, and metabotropic receptors. When physiological conditions are disturbed Zn^2+^ can be a very important factor which triggers the many cellular signaling processes leading to atrophy and cell death [14–17]. Based on clinical and preclinical studies, these molecular and cellular events associated with Zn^2+^ that have induced neurotoxicity may be linked with clinical cases of traumatic brain injury, stroke, epilepsy, Alzheimer's disease, and other neurodegenerative disorders [16, 18].

The first aim of this review is the presentation of the relationship between Zn^2+^, glutamate receptors, and transduction signaling pathways, leading to cell death in pathological conditions. The next purpose of this paper is to discuss the above foregoing molecular and cellular processes underlying brain disorders associated with cell death.

## 2. The Relationship between Zn^2+^ and the Glutamate System under Physiological Conditions

As we have already mentioned, the chelatable or free Zn^2+^ is solely localized within synaptic vesicles in the presynaptic terminals of glutamatergic neurons [9–11]. The intravesicular concentration of Zn^2+^ is provided by the vesicular zinc transporter, ZnT-3. However, the circumstances in which Zn^2+^ is released during synaptic activity and the physiological importance of released Zn^2+^ is not yet clear. The best-known postsynaptic target for released Zn^2+^ is the ionotropic glutamate N-methyl-D-aspartate receptors (NMDAR). Zn^2+^ shows a different affinity to these receptors, depending on its concentration. At a relatively low nanomolar concentration (10–20 nM) Zn^2+^ binds to a binding site localized on the GluN2A subunit of NMDAR, which allosterically inhibits the activity of the receptor [19]. It was postulated that this interaction may be responsible for tonic inhibition of NMDARs by ambient Zn^2+^ levels. If the concentration of Zn^2+^ is higher, and when it occurs at micromolar levels (IC_50_ ~ 50 *μ*m), Zn^2+^ binds to the GluN2B subunit of NMDAR, inhibiting NMDAR during phasic synaptic activity [20]. At a very high concentration of (10–50 *μ*M), Zn^2+^ in a voltage dependent manner blocks the channel of NMDAR [21]. The recent complex study of Vergnano et al., conducted on excitatory synapses from hippocampal mossy fibers-CA3 and Schaffer collateral-CA1, showed that the profile of Zn^2+^ action in glutamate synapses is closely related to the pattern of presynaptic activity and release probability. The results of these studies very seriously questioned the possibility of physiological tonic inhibition of GluN2A dependency because the tested synaptic Zn^2+^ concentration is very low (<10 nM). These findings are also related to mossy fibers-CA3 synapses, which are the most zinc-enriched synapses in the whole brain. However, these results do not exclude the potential role of ambient Zn^2+^ in the pathological states associated with glutamate over excitability, although this requires further studies being conducted. The next conclusion from this research concerns the modulation of NMDAR by Zn^2+^ in an activity dependent manner. Vergnano et al. in 2014 [22] showed that only repetitive stimulation of presynaptic terminals with proper frequency could result in the detectable higher Zn^2+^ concentration in the synaptic cleft. In contrast, a single stimulus was insufficient to achieve a similar synaptic event, thus indicating very efficient mechanisms responsible for Zn^2+^ removal from the synaptic space. Moreover, changes in the shape of excitatory postsynaptic potentials was observed which indicate Zn^2+^ binding to the nanomolar sensitive place of the GluN2A subunit on NMDARs [22]. Thus, it is questionable whether a GluN2B Zn^2+^ sensitive binding site may play a significant role in Zn^2+^-dependent modulation under physiological conditions.

## 3. Zn^2+^ Homeostasis

Because the Zn^2+^ level as well as the physiological norm may induce a different form of toxicity, the mechanisms that regulate concentration of extracellular and intracellular Zn^2+^ must act properly. The balance between extracellular, cytosolic and Zn^2+^ occurring in organelles is maintained by specific or nonspecific transporters and Zn^2+^ binding proteins. The most important regulators of Zn^2+^ level between cellular compartments, organelles, and extracellular space are membranous H^+^-Zn^2+^ exchangers that belong to the ZnT family [23]. The second group of Zn^2+^ transporters consists of members of the ZIP protein family that allows Zn^2+^ transportation from extracellular space or from intracellular vesicles to the cytoplasm [24]. So far, 10 members of the ZnT family and 14 members of the ZIP protein family have been identified [24]. The third group of molecules engaging in the homeostasis of Zn^2+^ is metallothioneins (MTs), which are low-weight molecular protein. The major cellular function of MTs is binding metals. Because MTs have a high affinity to Zn^2+^, they buffer cytoplasmic Zn^2+^ following its influx to cytosol. Furthermore, MTs play the role of a temporal store for cellular Zn^2+^. In physiological conditions, the foregoing mechanisms are sufficient to maintain the concentration of Zn^2+^ in a nontoxic range [16, 25]. Unfortunately, during a pathological condition, when excessive glutamate transmission occurs, the Zn^2+^ homeostatic system may become inefficient, leading to an increased intracellular level of Zn^2+^ and a subsequent activation of signaling pathways associated with cell death. These hypotheses concerning potential involvement of Zn^2+^ have numerous confirmations in studies using imaging techniques and selective ion chelators. The results obtained in these studies allowed understanding more adequately the importance of Zn^2+^ in the cellular mechanisms governing cell survival and forced researchers to revise the main assumptions concerning the molecular hypothesis of calcium ions (Ca^2+^) dependent excitotoxicity. For many years it was thought that intracellular calcium ions (Ca^2+^) are the most important factors inducing ischemic cell death. Most of these findings are based on experiments using imaging technics with Ca^2+^ sensitive fluorescent probes or Ca^2+^ selective chelators. Unfortunately, in the last years, it has been shown that these probes and chelators were not sufficiently selective, because many of them bind also Zn^2+^; thus the interpretation of these results may raise doubts [11]. Recently, Stork and Li 2006 showed that rat hippocampal slices subjected to oxygen glucose deprivation (OGD) and exposed to the cell-permeable Zn^2+^ chelator TPEN (low affinity for Ca^2+^) decreased fluorescence of low-affinity Ca^2+^ sensitive probe calcium green I during and after OGD. These results suggest that, at least in some circumstances, we can observe interplay between Ca^2+^ and Zn^2+^ in the induction of molecular consequences of OGD [26]. The direct involvement of Zn^2+^ in ischemia has been also shown in another study using TPEN. Medvedeva et al. in 2009 showed that increase in the concentration of intracellular Ca^2+^ in CA1 neurons in hippocampal slices observed a few minutes after OGD may be prevented by TPEN, which is a Zn^2+^ chelator. What is important is that the early increase in intracellular Zn^2+^ level is associated with mitochondrial Zn^2+^ uptake and depolarization, events which are engaged in neuronal cell death [27].

## 4. Zn^2+^, Glutamate Receptors, and the Signaling Pathway Leading to Cell Death—An Overview

The most neurotoxic effects induced by Zn^2+^ are a consequence of the impairment of Zn^2+^ homeostasis and the following increase of the postsynaptic cellular Zn^2+^ level. These pathological events have been reported in models of seizure, ischemic brain injury, or traumatic brain injury [28–30]. This raises a question concerning how Zn^2+^ can enter the cell under pathological conditions and, in turn, which signaling pathway activates it? It is well established that at least four different routes are involved in the increased Zn^2+^ cellular level. Two of them are, respectively, related to the Na^+^/Zn^2+^ exchanger and voltage gated calcium channels (VGCC). But the other two are associated with enhanced activity of ionotropic glutamate receptors. As has been reported in several studies, both AMPA/kainate and NMDA receptors are involved in Zn^2+^ entering postsynaptic terminals [31–35]. It seems that GluA2-lacking AMPA (*α*-amino-3-hydroxy-5-methyl-4-isoxazole propionic acid) receptors play a particularly important role in Zn^2+^ influx. These receptors are permeable for Zn^2+^ and Ca^2+^ ions, which after entering the cell activate signaling pathways involved in cell death. The significance of GluA2-lacking AMPAR in the induction of cell death of CA1 hippocampal neurons in transient global ischemia (TGI) and oxygen glucose deprivation (OGD) models has been reported. Inhibition of these receptors exerted neuroprotective effects [33, 36]. GluA2-lacking AMPARs seem to be very sensitive to extracellular acidosis, which is accompanied by ischemic conditions. The increased concentration of H^+^ ions invokes different effects on the permeability of these molecules to Zn^2+^ and Ca^2+^. Acidosis facilitates increased Zn^2+^ influx through GluA2-lacking AMPARs and VGCC. By contrast, in the same conditions, Ca^2+^ influx is decreased [37, 38]. Furthermore, it has been found that synaptic Zn^2+^ in the mossy fibers inhibits kainate receptor activity. If the pH is diminished, the Zn^2+^ effect on kainate receptors is abolished [39, 40]. Interplay between activity of the glutamate receptors, Zn^2+^, and acidosis is more complex, because Zn^2+^ itself may change its acid-based equilibrium. In cortical neurons Zn^2+^ leads to intracellular acidification [41]. Moreover, diminished intracellular pH attenuates Zn^2+^ binding to MTs and causes an increased level of the free form of this ion, which may promote the signaling pathway responsible for cell death [11].

**Figure 1:**
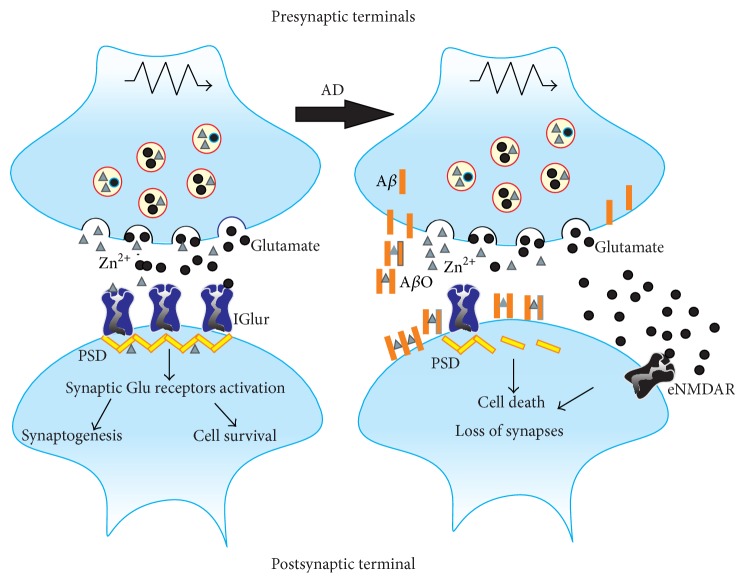
Intracellular signaling pathways activated by increased intracellular level Zn^2+^; MT: metallothioneins; ATP: adenosine triphosphate; ROS: reactive oxygen species; NO: nitric oxide.

AMPA/kainate receptors are not the only ones involved in the neurotoxic effects induced by Zn^2+^. The possible role of NMDA in Zn^2+^-induced neurotoxicity may be biphasic. As has been shown in cortical culture neurons, in the first step, the higher level of Zn^2+^ released from the presynaptic terminal may inhibit postsynaptic NMDAR function by allosteric binding. Subsequently, NMDAR upregulation induced by Src kinase mediated phosphorylation has been observed [42]. Zn^2+^ entrance into the postsynaptic terminal and then the increasing level of this ion are the first steps on the path to cell death. However, Zn^2+^ influx by glutamate ionotropic receptors or VGCCs is not a necessary condition to achieve the increased toxic intracellular level of Zn^2+^. The results of a few studies showed that mice lacking the gene encoding ZnT-3, which is responsible for loading Zn^2+^ to synaptic vesicles, have an increased intracellular level of Zn^2+^. Furthermore, signaling pathways and cell death have also occurred. It suggests that Zn^2+^ toxicity might be triggered by the release of this ion from its intracellular stores [43]. Whatever the cause, intracellular free Zn^2+^ interacts with many intracellular proteins and activates numerous processes, which in many ways damage cell structures and ultimately lead to their death. The toxic concentration of intracellular Zn^2+^ is especially averse to the functioning of mitochondria. Zn^2+^ is taken up in mitochondria by the activation of the cation-permeable channel, which is the mitochondrial Ca^2+^ uniporter. Enhanced sequestration of Zn^2+^ by mitochondria is observed under excitotoxic conditions. When intramitochondrial Zn^2+^ accumulation is sufficiently large, the membrane potential of mitochondria is disturbed and production of reactive oxygen species (ROS) is enhanced (Figure 1). These cellular events are very important factors in cell death [32, 41, 44]. It is worth mentioning that Ca^2+^ induced mitochondrial failure takes a similar course. The accumulation of Zn^2+^ in mitochondria also promotes the release of proapoptotic proteins such as apoptosis inducing factor (AIF) and cytochrome c [45, 46]. Additionally, Zn^2+^ may impair mitochondrial function by activation of the RAS/ERK/MAPK signaling pathway. Ultimately, the activation of MAPK induces mitochondrial hyperpolarization [47]. Furthermore, mitochondria are the main target of synergistic toxic effects induced by Zn^2+^ and kainate in the culture of cerebral cortex neurons [45, 46].

However, the dysfunction of mitochondria is not the only mechanism involved in Zn^2+^ toxic effect. Zn^2+^ overload observed in ischemic conditions disrupts the life processes of neurons in several different ways, including induction of oxidative stress and impairment of metabolism and, thus, production of ATP. The increase of the intracellular level of Zn^2+^ causes an enhanced activity of protein kinase C (PKC), which in turn activates NADPH oxidase. Hyperactivity of NAPDH determines the enhanced production of reactive oxygen species (ROS) [48, 49]. Furthermore, in cortical neurons, Zn^2+^ activates nitric oxide synthase (NOS), an essential enzyme for producing a very reactive and toxic peroxynitrite radical [48]. Zn^2+^ impairs metabolic processes by inhibiting glyceraldehydes-3-phosphate (GAPDH), which is the consequence of a reduction of cellular levels of NAD^+^ induced by Zn^2+^. Abolition of GAPDH metabolic function leads to a depletion of ATP and necrotic death of cells [50]. The effects of a reduced level of NAD^+^ are more complex on the cellular level of ATP and do not depend only on the inhibition of glycolytic enzymes. The decrease of NAD^+^ also inhibits activity of mitochondrial enzymes that are NAD^+^-dependent, such as *α*-ketoglutarate dehydrogenase or isocitrate dehydrogenase, and necessary for respiratory cellular processes [51].

## 5. Zn^2+^, Glutamate System, and Alzheimer's Disease

Expression of Alzheimer's disease (AD) is associated with several pathological features. Of particular importance in the development of this disease is its being attributed to the amyloid beta (A*β*) soluble oligomers, amyloid plaques deposit, and aggregation of neurofibrillary tangles (NFTs) containing a hyperphosphorylated form of protein tau. Amyloid oligomers both soluble and insoluble are composed of amyloid *β*, about 40-amino acid long peptide produced from amyloid precursor protein (APP). However, recently, the generation of cognitive impairments is more importantly attributed to soluble A*β* oligomers than amyloid plaques or NFTs [52–54]. Both accumulations of A*β*'s different forms and aggregation of NFTs lead to synaptic loss and finally neuronal death. The main signaling mechanisms that are engaged in neuronal death induced by NFTs and A*β* are mostly apoptotic [53]. Zn^2+^ is involved in at least three crucial events associated with the development of AD. First, Zn^2+^ binds to the A*β* monomer and then allows aggregation of monomers of A*β* to soluble A*β* oligomers and next to insoluble A*β* plaques. Aggregation of NFTs proceeds in a similar way. Zn^2+^ binds to a tau protein, allowing the production of a tau complex. Additionally, in AD, Zn^2+^ participates in autophagic dysfunction and deregulation of intraneuronal calcium equilibrium [8, 53, 55]. All of these events are correlated to the activation of many different signaling pathways involved in neuronal deterioration. Despite the fact that this review concerns the relationship between Zn^2+^, the glutamate system, and signaling pathways engaged in neurodegenerative conditions, a description of the relationship between oxidative stress, Zn^2+^, and AD will be omitted. We want to now focus our attention on the importance of Zn^2+^ in the formation of A*β* complexes and the influence of A*β* soluble oligomers on glutamate dependent signaling pathways. As we have mentioned a few times, Zn^2+^ is stored in synaptic vesicles of some glutamate neurons. As a result of stimulation of these neurons glutamate and Zn^2+^ are simultaneously released to a synaptic cleft [12, 17]. Additionally, stimulation of glutamate neurons causes the release of A*β* monomers from presynaptic terminals to a synapse. Studies conducted on hippocampal slices of rats and mice showed that an increased level of A*β* oligomers in the vicinity of the postsynaptic terminal is the final effect of simultaneously increasing levels of these molecules in the synaptic cleft. A*β* monomers are colocalized with NMDARs containing GluN2B subunits and a blockade of these receptors by nonselective NMDAR antagonist memantine, or GluN2B subunit selective antagonist ifenprodil, inhibits A*β* oligomers' localization in postsynaptic terminals [56].

**Figure 2:**
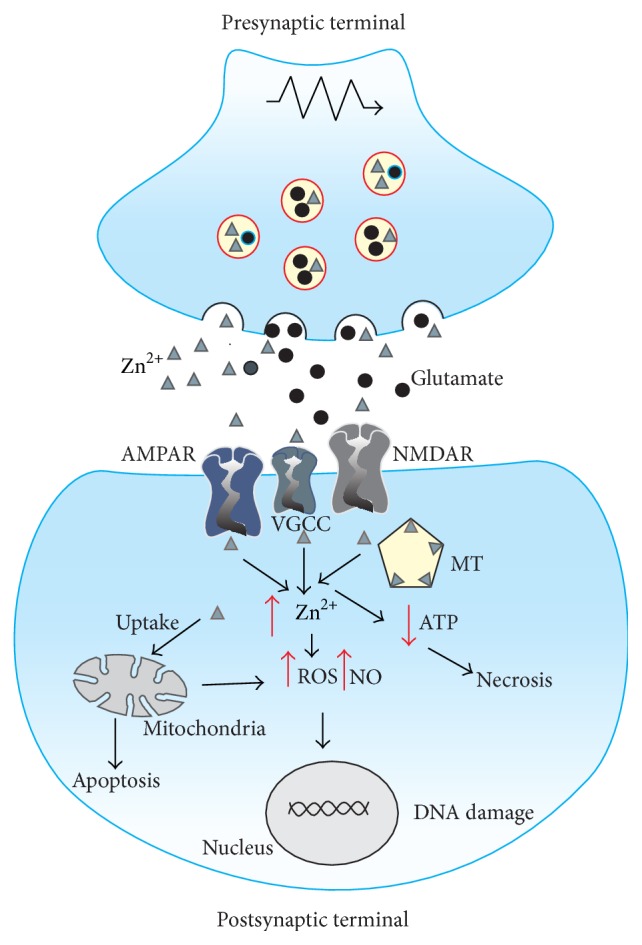
Synaptic interaction between Zn^2+^ and glutamate system observed in Alzheimer's disease (AD); IGlur (ionotropic glutamate receptors); PSD: postsynaptic density; A*β*: Amyloid *β*; A*β*O: Amyloid *β* oligomers.

Oligomerization of A*β* was also prevented by the addition of Zn^2+^-biding 8-OH-quinoline (clioquinol), by the lack of Zn^2+^ in the synapse, and by blockade of synaptic transmission. The role of Zn^2+^ in these processes relies on enabling the oligomerization of A*β* monomers and targeting A*β* oligomers to NMDARs containing GluN2B subunits [56]. However, it is unclear whether both of these processes (stabilizing oligomers and targeting of postsynaptic terminals) are involved in the synaptic events that are finally involved in the formation of cognitive impairments affecting patients with AD. But recent studies performed by Bjorklund et al. in 2012 [57] may bring a solution to this question. These postmortem studies have shown significant molecular differences between patients with AD and patients who have neuropathological changes in the absence of cognitive impairment, subsequently named nondemented with Alzheimer's neuropathology (NDAN). However, both groups of patients had higher levels of A*β* plaque and NFTs in the hippocampus compared to the control. Furthermore, soluble low molecular weight (LMW) A*β* oligomeric species were in these two groups at a similar level but significantly increased when compared to the control group. The main difference between the AD group and NDAN was the significantly higher level of LMW A*β* in the postsynaptic density (PSD) of the hippocampus of AD patients. Importantly, the level of synaptic LMW A*β* in the NDAN group was at a similar level when compared to the control. Both AD and NDAN groups had an increased level of Zn^2+^ in soluble fraction compared to the control, but the level of Zn^2+^ in AD was also significantly higher in comparison to NDAN. Vesicular Zn^2+^ was at a similar level in the two groups, but in the AD group the level of ZnT-3 was declined. The last distinction between AD and NDAN seems to be particularly important, because it shows a decreased level of the phosphorylated form of the cAMP response element binding (CREB) in CA3 and dentate gyrus of the hippocampus of AD patients when compared to the NDAN group [57]. The role of CREB and its active phosphorylated form in the synaptogenesis and stability of synapses and then in processes of learning and memory has been reported in many previous studies [58–60]. Additionally, a decreased level of phospho-CREB and a higher level of LMW A*β* in the AD hippocampus may be associated with dysfunction and abnormalities in the glutamate system. Soluble A*β* oligomers inhibit LTP in hippocampal slices, and this effect was prevented by selective GluN2B subunit antagonists, such as ifenprodil and Ro-25-6981 [61, 62]. As mentioned earlier, ifenprodil also prevented binding soluble A*β* oligomers to the postsynaptic terminal in the presence of Zn^2+^ in the synaptic cleft [56]. Furthermore, A*β* oligomers in hippocampal slices enhanced glutamate transmission through extrasynaptic NMDARs containing a GluN2B subunit with concomitant activation of proapoptotic p38 mitogen-activated kinase (MAPK) and decreased phosphorylation of CREB. Selective NMDA antagonists prevented activation of MAPK and reduction of CREB phosphorylation [62]. Furthermore in their previous studies Li et al. also showed that A*β* oligomers in hippocampal neurons lead to long-term depression [63]. In other studies, A*β* oligomers activate glial *α*7 nicotinic acetylcholine receptors, which results in astrocytic glutamate and activation of extrasynaptic glutamate receptors. These mechanisms indicate an enhanced glutamate transmission via extrasynaptic receptors versus synaptic transmission. This finally results in glutamate excitotoxicity and subsequent cell death [64] (Figure 2). The involvement of NMDAR in pathological processes underlying AD suggests that NMDAR antagonists may be effective drugs in the treatment of AD. Unfortunately, the results obtained in clinical practice must subdue these hopes. The administration of memantine, currently approved NMDAR antagonist in AD, has some restrictions. Memantine in a few clinical trials showed little improvements of cognitive functions and mood in moderate to severe AD. However, the clinical efficacy of memantine in the treatment of mild to moderate AD is still unknown. In contrast to another NMDAR antagonist like ketamine, memantine is well-tolerated drug and the adverse effects are not too severe. Opportunities for more effective drugs to treat AD are derivatives of memantine [65]. When considering the involvement of Zn^2+^ in processes concerning the oligomerization of A*β*, as in both the results obtained by Bjorklund et al. in 2012 [57] and conclusions from previous studies indicating the relationship between A*β* oligomers and glutamate transmission through extrasynaptic NMDARs, it is plausible to postulate Zn^2+^ participation in glutamate's excitotoxicity, which is an underlying cognitive dysfunction observed in AD. However, in order to know the precise importance of Zn^2+^ in glutamate dependent signaling in AD, further and more detailed studies are required. First of all, there is a lack of sufficiently clear evidence that Zn^2+^ plays the main role in the oligomerization of toxic A*β* soluble oligomers in the pathogenesis of AD. Several studies also showed that Cu^2+^ engaged in the oligomerization of A*β*. Furthermore, results obtained from in vitro studies show that Cu^2+^ in a specific manner stabilize A*β* oligomer structures and these, but not Zn^2+^, containing A*β* oligomers are toxic to neuronal cells [66]. We cannot exclude that the toxicity of A*β* oligomers during AD is the result of interplay between Cu^2+^ and Zn^2+^ availability in synapses. The next unsolved issue concerns the accumulation of Zn^2+^ in amyloid plaques. It is well known that increased Zn^2+^ binding to amyloid plaque reduces Zn^2+^ presence in the synaptic vesicle and then indirectly affects glutamatergic transmission. Absorption of Zn^2+^ by A*β* oligomers may lead to disruption of the glutamate transmission and dysregulation of ProSAP2/11 Shank3 scaffolding [67]. Briefly, in normal condition, ProSAP2/Shank3 proteins are responsible for formulating scaffold in postsynaptic density. Properly formulated ProsSAP2/Shank3 complexes play a central role in the structure of the synapse and allow for the incorporation of other key proteins in postsynaptic density. Formation of ProSAP2/Shank3 is dependent on local Zn^2+^ concentration and influx. As has been reported by Grabrucker and colleagues in 2014 [67], application of A*β* oligomers in rat hippocampal cultures prevented normal association of Zn^2+^ with ProSAP/Shank3 and significantly decreased the Zn^2+^ level in dendrites. These changes were accompanied by decreased levels of Shank 1 protein in PSD and decreased synapse density (Figure 2). Interestingly, Zn^2+^ deficiency and deficits in the normal functioning of synapses associated with the dysfunction of ProSAP/Shank protein were also observed in autism [68]. AD affects mainly the elderly, and such patients have a reduced level of the vesicular ZnT-3 transporter, which can be the next factor reducing the pool of vesicle Zn^2+^ [69]. When we take into account relationship between Zn^2+^, glutamate transmission, and AD, the situation becomes even more complicated. The proper activity of glutamate neurons in many brain regions also involved in AD phenotype is crucial factor in the equilibrium between LTP and LTD processes which underlies synaptogenesis and neuronal death or survival [70]. At molecular level, these processes are dependent on suitable level of neurotrophins and on the involvement of these proteins in synaptic activity [70]. The results obtained in many postmortem studies in AD patients indicated decreased level of neurotrophin like brain derived neurotrophic factor (BDNF) (decreased level of mRNA and pro-BDNF (precursor of BDNF) and mature BDNF) and neural growth factor (NGF) (increased level of NGF precursor (pro-NGF) and decreased level of NGF) in brain regions like basal forebrain or hippocampus whose functions are impaired in AD. As has been shown in preclinical studies the effect of Zn^2+^ on the expression of these proteins is varied and ambiguous [71]. On one hand, studies conducted by Corona et al. in 2010 showed that Zn^2+^ supplementation in AD transgenic mice significantly delays hippocampal-dependent memory deficits and diminishes both A*β* and tau pathology in the hippocampus. These changes are associated with increased expression of BDNF synthesis [72], which may confirm previously described potential of Zn^2+^ to BDNF maturation from pro-BDNF through activation of Zn^2+^-dependent matrix metalloproteinases (MMPs) [73]. In contrast to that, Linkous et al. in 2009 showed that Zn^2+^ supplementation leads to cognitive deficits in AD transgenic mice. These equivocal effects of Zn^2+^ supplementation on cognitive capacities observed in transgenic mice can be explained as a consequence of two different AD transgenic models used by these two research groups. Corona and colleagues used 3xTg-AD mice with AD phenotype predominantly associated with intraneuronal deposition of A*β*. Linkous and his colleagues conducted their experiments on Tg2576 and TgCRND mice manifest memory deficits related to extracellular A*β* overload [74]. These facts generate the additional questions about the role of Zn^2+^, neurotrophins, and development in AD. Furthermore it is very possible that binding Zn^2+^ to NGF molecule is required to achieve full biological properties [71]. The complexity of the relationship between Zn^2+^, AD, and glutamate transmission does not allow for the exclusion of all these factors involved in the development of AD during different stages of the disease. Thus, both reduced and increased concentrations of Zn^2+^ in AD, depending on the stage of the disease and the structure of the brain, can have positive or negative effects on the symptomatic expression of AD. The role of Zn^2+^ in pathophysiology of AD seems to be emphasized by the results of studies using PBT2 which is the Cu^2+^/Zn^2+^ ionophore. The mechanism of action of PBT2 involves liberation of Cu^2+^ and Zn^2+^ from complexes of A*β*. In animal models of AD administration PBT2 leads to clearance of A*β* aggregates which markedly improves cognitive function observed in behavioral tests. The potential efficacy of this compound in the treatment in AD was confirmed in Phase II, double-blind, randomized, placebo controlled trial. In these studies the oral administration of 250 mg of PBT2 by 12 weeks was well tolerated by patients. The analysis of cerebrospinal fluid showed the decreased level of A*β*. These events were correlated with improved executive functions measured in neuropsychological test battery [75].

## 6. Conclusions

As we have tried to show in the present paper, an increased amount of evidence indicates a relationship between abnormalities in the functioning of the glutamate system and development of neurodegenerative processes. However, a precise molecular set of causes leading to the dysfunction of glutamate pathways is still insufficiently understood. The results obtained in many studies indicate at least a mutual relationship between Zn^2+^ level fluctuations in nerve cells and structural and functional dysfunction of glutamate neurons containing Zn^2+^. The involvement of Zn^2+^ in the oxidative stress processes observed in ischemic neurons is well established and is related to enhanced activity of glutamate transmission. Furthermore, studies concerning the pathogenesis of Alzheimer's disease showed that Zn^2+^ is engaged in many ways to the potentiation of toxic effects invoked by glutamate. Importantly, some results obtained from different models are corroborated in postmortem studies. Despite the undoubted successes in the understanding of Zn^2+^ important role in the functioning of many aspects of the glutamate system, a global perspective still remains lacking. For example, both Alzheimer's disease and brain injury caused by ischemia may have their own time course. Thus, further studies concerning the pathological role of Zn^2+^ caused/induced by glutamate toxicity should attempt to explain the precise role of Zn^2+^ in different stages of these disorders.
